# Author Correction to: Prevalence of Use and Cost of Biological Drugs for Cancer Treatment: A 5-Year Picture from Southern Italy

**DOI:** 10.1007/s40261-017-0613-1

**Published:** 2017-12-30

**Authors:** Simona Lucchesi, Ilaria Marcianò, Paolo Panagia, Rosanna Intelisano, Maria Pia Randazzo, Carmela Sgroi, Giuseppe Altavilla, Mariacarmela Santarpia, Vincenzo Adamo, Tindara Franchina, Francesco Ferraù, Paolina Reitano, Gianluca Trifirò

**Affiliations:** 1 Department of Chemical Sciences, Biological, Pharmaceutical and Environmental, University of Messina, Messina, Italy; 2 Clinical Pharmacology Unit, A.O.U. Policlinico “G. Martino”, Messina, Italy; 3 A.O.U. Policlinico “G. Martino”, Messina, Italy; 4 Papardo Hospital, Messina, Italy; 5 Pharmaceutical Department, Local Health Unit of Messina, Messina, Italy; 6 Medical Oncology Unit, Department of Adult and Childhood Human Pathology “G. Barresi”, University of Messina, Messina, Italy; 7 Medical Oncology Unit, Papardo Hospital, Messina, Italy; 8 Medical Oncology Unit, Hospital “San Vincenzo”, Messina, Taormina Italy; 9 Department of Biomedical and Dental Sciences and Morphofunctional Imaging, University of Messina, Via Consolare Valeria, Messina, Italy; 10 Department of Medical Informatics, Erasmus Medical Center, Rotterdam, The Netherlands

## Author Correction to: Clin Drug Investig https://doi.org/10.1007/s40261-017-0591-3

Unfortunately, many errors were identified in the published article. The original article was corrected.

**Article Title**, which previously read:

Prevalence of Use and Cost of Biological Drugs for Cancer Treatment: A 5-Year Picture from Southern Italy

Should read:

Prevalence of Use and Cost of Biological and Non-Biological Targeted Therapies for Cancer Treatment: A 5-Year Picture from Southern Italy

**Article running header**, which previously read:

5-Year Prevalence of Use and Costs of Biologics in Oncology in Southern Italy

Should read:

Cost of Biological and Non-Biological Targeted Therapies in Oncology in Southern Italy

**Abstract: Background and Objectives**, which previously read:

Considering the clinical and economic burden of biological drugs in cancer treatment, it is necessary to explore how these drugs are used in routine care in Italy and how they affect the sustainability of the National Health Services. This study aimed to investigate the prevalence of use and costs of biological drugs for cancer treatment in a general population of Southern Italy in the years 2010–2014.

Should read:

Considering the clinical and economic burden of biological and non-biological targeted therapies in cancer treatment, it is necessary to explore how these drugs are used in routine care in Italy and how they affect the sustainability of the National Health Services. This study aimed to investigate the prevalence of use and costs of biological and non-biological targeted therapies for cancer treatment in a general population of Southern Italy in the years 2010–2014.

**Abstract: Methods, 2nd sentence**, which previously read:

In this study, users of biological drugs for cancer treatment were characterized and the prevalence of use and costs were calculated over time.

Should read:

In this study, users of biological and non-biological targeted therapies for cancer treatment were characterized and the prevalence of use and costs were calculated over time.

**Abstract: Conclusions**, which previously read:

In recent years, the use and costs of biological drugs in cancer patients dramatically increased in a large population from Southern Italy. This trend may be counterbalanced by adopting biosimilars once they are available. Claims databases represent a valid tool to monitor the uptake of newly marketed biological drugs and biosimilars.

Should read:

In recent years, the use and costs of biological and non-biological targeted therapies in cancer patients dramatically increased in a large population from Southern Italy. This trend may be counterbalanced by adopting biosimilars once they are available. Claims databases represent a valid tool to monitor the uptake of newly marketed biological drugs and biosimilars as well as other non-biological targeted therapies.

**Key Points, 1st Key Point**, which previously read:

In recent years, the use of biological drugs for cancer treatment rapidly increased and the corresponding costs almost doubled from €6.6 to €13.6 million.

Should read:

In recent years, the use of biological and non-biological targeted therapies for cancer treatment rapidly increased and the corresponding costs almost doubled from €6.6 to €13.6 million.

**Key Points, 3rd Key Point**, which previously read:

Claims databases may represent a valid tool for monitoring the uptake of newly marketed biological drugs and biosimilars.

Should read:

Claims databases may represent a valid tool for monitoring the uptake of newly marketed biological drugs and biosimilars as well as other innovative targeted therapies.

**Section 1, 2nd paragraph, 1st sentence**, which previously read:

Considering the clinical and economic burden of biological drugs in cancer treatment, it is necessary to explore how these drugs are used in routine care and how they affect the sustainability of the National Health Services (NHSs).

Should read:

Considering the clinical and economic burden of biological and non-biological targeted therapies in cancer treatment, it is necessary to explore how these drugs are used in routine care and how they affect the sustainability of the National Health Services (NHSs).

**Section 1, Introduction, 4th paragraph, 1st sentence**, which previously read:

The aim of this retrospective, observational study was to analyze the use and costs of biologic drugs for cancer treatment in a large area of Southern Italy in the years 2010–2014.

Should read:

The aim of this retrospective, observational study was to analyze the use and costs of biological and non-biological targeted therapies for cancer treatment in a large area of Southern Italy in the years 2010–2014.

**Section 2.1, 1st paragraph, last line**, which previously read:

Each of these centers provided information on the total use of biological drugs for cancer treatment from all residents in Messina Province (Southern Italy).

Should read:

Each of these centers provided information on the total use of biological and non-biological targeted therapies for cancer treatment from all residents in Messina Province (Southern Italy).

**Section 2.1, 2nd paragraph, 3rd, 4th and sentences**, which previously read:

In outpatients, systemic biological drugs administered as subcutaneous injections or orally are dispensed by the hospital pharmacists to the patient, who will self-administer the drug. Systemic biological drugs administered as an intravenous infusion are administered exclusively in the hospital setting, even to outpatients. However, the dispensing of biological drugs to outpatients is recorded at patient level through the dispensing database, which is routinely populated by the hospital pharmacy.

Should read:

In outpatients, systemic targeted therapies administered as subcutaneous injections or orally are dispensed by the hospital pharmacists to the patient, who will self-administer the drug. Systemic targeted therapies administered as an intravenous infusion are administered exclusively in the hospital setting, even to outpatients. However, the dispensing of biological and non-biological targeted therapies to outpatients is recorded at patient level through the dispensing database, which is routinely populated by the hospital pharmacy.

**Section 2.3, 1st sentence**, which previously read:

The biological drugs approved for cancer treatment and available in Italy during the study years were classified into mAbs, fusion proteins, immunomodulatory agents, and small molecules, the latter being further categorized as TKIs, mammalian target of rapamycin inhibitors (mTOR-i), and proteasome inhibitors.

Should read:

The biological and non-biological targeted therapies approved for cancer treatment and available in Italy during the study years were classified into mAbs, fusion proteins, immunomodulatory agents, and small molecules, the latter being further categorized as TKIs, mammalian target of rapamycin inhibitors (mTOR-i), and proteasome inhibitors.

**Section 2.4, 2nd paragraph, last line**, which previously read:

In addition, the pharmaceutical expenditure for the study drugs was measured over time and stratified by type of biological drug.

Should read:

In addition, the pharmaceutical expenditure for the study drugs was measured over time and stratified by type of drug.

**Section 2.4, 3rd paragraph, 1st sentence**, which previously read:

Users of different types of biological drug were characterized in terms of age and sex, type of cancer, and previous use of chemotherapeutics.

Should read:

Users of different types of study drugs were characterized in terms of age and sex, type of cancer, and previous use of chemotherapeutics.

**Table 1**: There were errors in Table 1, below is the correct version of Table 1:

**Table 1:** Characteristics of users of monoclonal antibodies and other non-biological targeted therapies (i.e. small molecules) for cancer treatment in the years 2010–2014 in Messina Province

Characteristic	mAbs (*n* = 1607)	Small molecules				Total (*n* = 2491)
		TKIs (*n* = 609)	Proteasome inhibitors (*n* = 203)	mTOR-i (*n* = 72)	Total (*n* = 884)	
Sex						
Male	638 (39.7)	382 (62.7)	95 (46.8)	21 (29.2)	498 (56.3)	1136 (45.6)
Female	969 (60.3)	227 (37.3)	108 (53.2)	51 (70.8)	386 (43.7)	1355 (56.4)
Age (years) [median (Q1–Q3)]	62 (53–71)	65 (56–74)	70 (61–77)	63 (54.5–71.5)	67 (58–75)	64 (54–72)
Age categories (years)						
< 45	158 (9.8)	44 (7.2)	3 (1.5)	4 (5.6)	51 (5.7)	209 (8.4)
45–64	759 (47.2)	246 (40.4)	60 (29.6)	35 (48.6)	341 (38.6)	1100 (44.2)
65–79	589 (36.7)	265 (43.5)	113 (55.7)	26 (36.1)	404 (45.7)	993 (39.9)
≥ 80	101 (6.3)	54 (8.9)	27 (13.3)	7 (9.7)	88 (10.0)	189 (7.5)
Follow-up (days) [median (Q1–Q3)]	327 (130–595)	313 (91–867)	320 (132–644)	225 (69–358.5)	305 (95.5–777)	319 (119–640)
Number of dispensing of the study drugs at ID [median (Q1–Q3)]	7 (3–14)	4 (2–12)	16 (8–25)	3 (1–6)	5 (2–16)	6 (3–14)
Type of cancer^a^						
Lymphatic tissue^b^	268 (16.7)	2 (0.3)	3 (1.5)		5 (0.6)	273 (11.0)
Breast (female)	220 (13.7)	10 (1.6)		4 (5.6)	14 (1.6)	234 (9.4)
Colorectal	148 (9.2)	3 (0.5)			3 (0.3)	151 (6.1)
Leukemia	77 (4.8)	84 (13.8)			84 (9.5)	161 (6.5)
Lung	24 (1.5)	79 (13.0)			79 (8.9)	103 (4.1)
Liver cancer	5 (0.3)	48 (7.9)			48 (5.4)	53 (2.1)
Multiple myeloma	4 (0.2)		116 (57.1)		116 (13.1)	120 (4.8)
Metastasis of unspecified primary tumor	389 (24.2)	102 (16.7)	1 (0.5)	8 (11.1)	111 (12.6)	500 (20.1)
Other types of cancer^c^	124 (7.7)	55 (9.0)	14 (6.9)	5 (6.9)	74 (8.4)	198 (7.9)
Not reported	348 (21.7)	226 (37.1)	69 (34.0)	55 (76.4)	350 (39.6)	698 (28.0)
Previous chemotherapy^d^						
Number of chemotherapeutics						
0	916 (57.0)	517 (84.9)	193 (95.1)	34 (47.2)	744 (84.2)	1660 (66.6)
1	220 (13.7)	49 (8.0)	9 (4.4)	34 (47.2)	92 (10.4)	312 (12.5)
2–3	422 (26.3)	42 (6.9)	1 (0.5)	4 (5.6)	47 (5.3)	469 (18.9)
≥4	49 (3.0)	1 (0.2)			1 (0.1)	50 (2.0)
Type of chemotherapeutics						
Cyclophosphamide	342 (21.3)	1 (0.2)	1 (0.5)		2 (0.2)	344 (13.8)
Fluorouracil	234 (14.6)	1 (0.2)		1 (1.4)	2 (0.2)	236 (9.5)
Doxorubicin	153 (9.5)		7 (3.9)	4 (5.6)	11 (1.2)	164 (6.6)
Epirubicin	161 (10.0)	1 (0.2)			1 (0.1)	162 (6.5)
Docetaxel	128 (8.0)	17 (2.8)		2 (2.8)	19 (2.1)	147 (5.9)
Vincristine	99 (6.2)		2 (1.0)		2 (0.2)	101 (4.1)
Oxaliplatin	71 (4.4)			1 (1.4)	1 (0.1)	72 (2.9)
Capecitabine	40 (2.5)	14 (2.3)		4 (5.6)	18 (2.0)	58 (2.3)
Paclitaxel	51 (3.2)	1 (0.2)		3 (4.2)	4 (0.5)	55 (2.2)
Gemcitabine	12 (0.7)	34 (5.6)		2 (2.8)	36 (4.1)	48 (1.9)
Vinorelbine	14 (0.9)	23 (3.8)		7 (9.7)	30 (3.4)	44 (1.8)
Carboplatin	17 (1.1)	24 (3.9)		1 (1.4)	25 (2.8)	42 (1.7)
Triptorelin	32 (2.0)	5 (0.8)		2 (2.8)	7 (0.8)	39 (1.6)
Fulvestrant	19 (1.2)			10 (13.9)	10 (1.1)	29 (1.2)
Bendamustine	27 (1.7)					27 (1.1)
Fludarabine	25 (1.6)					25 (1.0)
Others^e^	54 (3.4)	24 (3.9)	2 (1.0)	6 (8.3)	32 (3.6)	86 (3.5)

Data are given as *n* (%) unless otherwise specified

Patients (*n* = 8) who were dispensed two different drugs at the index date were excluded

Patients (*n* = 2) whose sex and age were not available were excluded

No users of fusion proteins or immunomodulatory agents could be identified during the study years, and these two drug categories are therefore not included

*ID* index date, *mAb* monoclonal antibodies, *mTOR-i* mammalian target of rapamycin inhibitors, *Q1–Q3* interquartile range, *TKIs* tyrosine-kinase inhibitors

^a^Type of cancer refers to the last cancer diagnosis registered within 6 months prior to the first dispensing of the study drugs, during the study period

^b^Neoplasms of lymphatic tissue include lymphosarcoma and reticulosarcoma, Hodgkin’s disease, non-Hodgkin’s lymphoma

^c^Other neoplasms include neoplasms of peritoneum, eye, brain, thyroid, bones and connective tissue, genitourinary system, pancreas, respiratory organs (other than lungs), skin, carcinomas in situ, monoclonal gammopathy, prostate, benign neoplasm, breast (males), bladder and kidney, esophagus, stomach, duodenum, trachea, larynx, nasal cavities and neoplasms of unspecified nature

^d^Chemotherapeutics were identified within 6 months prior to the first dispensing of the study drugs, during the study period

^e^Other chemotherapeutics include cisplatin, pemetrexed, vinblastine, temozolomide, bleomycin, dacarbarzine, methotrexate, etoposide, eribulin, topotecan, azacitidine, cabazitaxel, mitoxantrone, tegafur, vindesine, fotemustine

**Section 3, 3rd paragraph**, which previously read:

During the study years, the total prevalence of use of biological drugs for cancer treatment doubled from 0.9 (in 2010) to 1.8 (in 2014) per 1000 inhabitants, mostly due to the increased use of small molecules (+ 120.8%) rather than mAbs (+ 88.4%) (Fig. 1, Electronic Supplementary Material Table S2).

Should read:

During the study years, the total prevalence of use of study drugs for cancer treatment doubled from 0.9 (in 2010) to 1.8 (in 2014) per 1000 inhabitants, mostly due to the increased use of small molecules (+ 120.8%) rather than mAbs (+ 88.4%) (Fig. 1, Electronic Supplementary Material Table S2).

**Section 3, 4th paragraph**, which previously read:

Accordingly, the costs of the biological drugs for cancer treatment rapidly grew during the study years in Messina Province from €6.6 million in 2010 (*n* = 591) to €13.6 million in 2014 (*n* = 1150), with a total expenditure of around €50 million during the five observation years (Fig. 2). Likewise, the number of different biological drugs that were prescribed to the study population increased from 17 in 2010 to 21 in 2014 (data not shown).

Should read:

Accordingly, the costs of the biological and non-biological targeted therapies for cancer treatment rapidly grew during the study years in Messina Province from €6.6 million in 2010 (*n* = 591) to €13.6 million in 2014 (*n* = 1150), with a total expenditure of around €50 million during the five observation years (Fig. 2). Likewise, the number of different biological and non-biological targeted therapies that were prescribed to the study population increased from 17 in 2010 to 21 in 2014 (data not shown).

**Section 3, 5th paragraph, 1st sentence**, which previously read:

In 2020, based on our predictions, the expenditure for biological study drugs will grow to €25 million.

Should read:

In 2020, based on our predictions, the expenditure for monoclonal antibodies and other non-biological targeted therapies will grow to €25 million.

**Section 4, 1st paragraph**, which previously read:

To our knowledge, this is the first observational study investigating the prevalence of use and the costs of biological drugs in oncology in a large area of Southern Italy using administrative healthcare databases.

Should read:

To our knowledge, this is the first observational study investigating the prevalence of use and the costs of monoclonal antibodies and other non-biological targeted therapies in oncology in a large area of Southern Italy using administrative healthcare databases.

**Section 4, 2nd paragraph**, which previously read:

Our results showed a dramatic increase in biological drug use in oncology, considering both mAbs and small molecules. These data are in line with the National Report on Medicines Use in Italy in 2015 [9], which described an 18.2% increase in mAb consumption (ATC I level: L) in comparison with the previous year. There may be different reasons to explain the increasing number of cancer patients using biological drugs. In recent years, an increasing number of biological drugs have been marketed in Italy, as confirmed by the increasing number of different ATCs for cancer treatment dispensed in Messina during the study years (from 17 in 2010 to 21 in 2014; data not shown). Furthermore, many biological drugs already approved for cancer treatment gained an extension to their indications of use, thus guaranteeing access to these innovative therapies to a larger number of patients. We observed an increase in the number of prevalent users over time, despite a decrease in the proportion of incident users (from 61.4% in 2011 to 54.4% in 2014; data not shown). These results reflect a growing number of patients taking biological drugs for a longer period of time, rather than an increase in those initiating treatment. During the study years, no users of fusion proteins or immunomodulatory agents could be identified. Specifically, use of aflibercept has only been approved in Sicily since November 2014 and we therefore could not identify any users of this drug. Due to their costs, many biological drugs in oncology are included among the top 30 molecules for drug expenditure sustained by public hospitals, with trastuzumab, bevacizumab, and rituximab being the top three.

Should read:

Our results showed a dramatic increase in biological and non-biological targeted therapies use in oncology, considering both mAbs and small molecules. These data are in line with the National Report on Medicines Use in Italy in 2015 [9], which described an 18.2% increase in mAb consumption (ATC I level: L) in comparison with the previous year. There may be different reasons to explain the increasing number of cancer patients using the study drugs. In recent years, an increasing number of biological and non-biological targeted therapies have been marketed in Italy, as confirmed by the increasing number of different ATCs for cancer treatment dispensed in Messina during the study years (from 17 in 2010 to 21 in 2014; data not shown). Furthermore, many biological and non-biological targeted therapies already approved for cancer treatment gained an extension to their indications of use, thus guaranteeing access to these innovative therapies to a larger number of patients. We observed an increase in the number of prevalent users over time, despite a decrease in the proportion of incident users (from 61.4% in 2011 to 54.4% in 2014; data not shown). These results reflect a growing number of patients taking monoclonal antibodies and other non-biological targeted therapies (small molecules) for a longer period of time, rather than an increase in those initiating treatment. During the study years, no users of fusion proteins or immunomodulatory agents could be identified. Specifically, use of aflibercept has only been approved in Sicily since November 2014 and we therefore could not identify any users of this drug. Due to their costs, many targeted therapies in oncology are included among the top 30 molecules for drug expenditure sustained by public hospitals, with trastuzumab, bevacizumab, and rituximab being the top three.

**Figure 1 legend**, which previously read:

Prevalence of biological drugs use for cancer treatment per 1000 inhabitants, stratified by calendar year. *mAb* monoclonal antibodies, *mTOR* mammalian target of rapamycin, *TKI* tyrosine kinase inhibitors

Should read:

Prevalence of biological and non-biological targeted therapies use for cancer treatment per 1000 inhabitants, stratified by calendar year. *mAb* monoclonal antibodies, *mTOR* mammalian target of rapamycin, *TKI* tyrosine kinase inhibitors

**Figure 2 legend**, which previously read:

Expenditure for the dispensing of biological drugs in oncology in Messina Province in the years 2010–2014, stratified by calendar year and type of biological drugs. *mAb* monoclonal antibodies, *mTOR-i* mammalian target of rapamycin inhibitors, *proteas-i* proteasome inhibitors, *TKI* tyrosine kinase inhibitors

Should read:

Expenditure for the dispensing of biological and non-biological targeted therapies in oncology in Messina Province in the years 2010–2014, stratified by calendar year and type of drug. *mAb* monoclonal antibodies, *mTOR-i* mammalian target of rapamycin inhibitors, *proteas-i* proteasome inhibitors, *TKI* tyrosine kinase inhibitors. The purple line indicates the number of patients receiving the study drugs

**Figure 3 legend**, which previously read:

Prevision of expenditure for biological drugs for cancer treatment in Messina area, assuming an uptake of trastuzumab and rituximab biosimilars of 0, 20, 50, and 80%

Should read:

Prevision of expenditure for biological and non-biological targeted therapies for cancer treatment in Messina area, assuming an uptake of trastuzumab and rituximab biosimilars of 0, 20, 50, and 80%

**Section 4, 4th paragraph**, which previously read:

The assumptions taken into account for the forecast of the expected expenditure on biological drugs in oncology until 2020 are as follows:

Should read:

The assumptions taken into account for the forecast of the expected expenditure on monoclonal antibodies plus other non-biological targeted therapies drugs in oncology until 2020 are as follows:

**Section 4, 7th paragraph, 3rd line**, which previously read:

On the other hand, the future marketing of innovative and highly priced biological drugs for the treatment of cancer will likely increase pharmaceutical expenditure.

Should read:

On the other hand, the future marketing of innovative and highly priced drugs for the treatment of cancer will likely increase pharmaceutical expenditure.

**Section 4, 7th paragraph, last line**, which previously read:

In addition, patients treated first with the study biological drugs or with the corresponding biosimilars may switch to newly marketed innovative drugs, thus leading to an increase in total expenditure and to a lower uptake of biosimilars.

Should read:

In addition, patients treated first with the study drugs or with the biosimilars may switch to newly marketed innovative drugs, thus leading to an increase in total expenditure and to a lower uptake of biosimilars.

**Section 4, 9th paragraph, 1st line**, which previously read:

In such a context, post-marketing monitoring systems using real-world data may allow rapid evaluations of the uptake, appropriate use, safety, and economic impact of the high-cost biological drugs and their corresponding biosimilars in cancer patients, thus optimizing pharmaceutical expenditure.

Should read:

In such a context, post-marketing monitoring systems using real-world data may allow rapid evaluations of the uptake, appropriate use, safety, and economic impact of the high-cost biological drugs and their corresponding biosimilars as well as other non-biological innovative targeted therapies in cancer patients, thus optimizing pharmaceutical expenditure.

**Section 4, 9th paragraph, 2nd line**, which previously read:

For most of the biological drugs approved for cancer treatment, the Italian Drug Agency implemented drug-specific monitoring registries as tools to monitor the appropriate use, effectiveness, and safety of those drugs that may facilitate post-marketing monitoring, although so far these registries have not been systematically used for scientific purposes [19].

Should read:

For most of the monoclonal antibodies and other non-biological targeted therapies approved for cancer treatment, the Italian Drug Agency implemented drug-specific monitoring registries as tools to monitor the appropriate use, effectiveness, and safety of those drugs that may facilitate post-marketing monitoring, although so far these registries have not been systematically used for scientific purposes [19].

**Section 4.1, 1st paragraph, 1st line**, which previously read:

Using administrative healthcare databases, including dispensing data and the hospital discharge diagnosis, this observational study investigated the prevalence of use and the costs of biological drugs in oncology in a large area from Southern Italy, covering a population of more than 650,000 people.

Should read:

Using administrative healthcare databases, including dispensing data and the hospital discharge diagnosis, this observational study investigated the prevalence of use and the costs of biological and non-biological targeted therapies in oncology in a large area from Southern Italy, covering a population of more than 650,000 people.

**Section 4.1, 1st paragraph, last line**, which previously read:

Due to the frequency of administration, especially for infusion biological drugs, patients are much more likely to choose the closest oncology center.

Should read:

Due to the frequency of administration, especially for infusion study drugs, patients are much more likely to choose the closest oncology center.

**Section 5, 1st line**, which previously read:

The use of and corresponding expenditure relating to biological drugs for cancer treatment has rapidly and dramatically increased, almost doubling over a 5-year period in a large general population of Southern Italy.

Should read:

The use of and corresponding expenditure relating to monoclonal antibodies and other non-biological targeted therapies for cancer treatment has rapidly and dramatically increased, almost doubling over a 5-year period in a large general population of Southern Italy.

**Section 5, last line**, which previously read:

On the other hand, real-world data are essential to rapidly monitor the benefit–risk profile and appropriate use of biological drugs and related biosimilars in routine care, with the final goal being to optimize pharmaceutical expenditure in oncology patients.

Should read:

On the other hand, real-world data are essential to rapidly monitor the benefit–risk profile and appropriate use of monoclonal antibodies and related biosimilars as well as other non-biological targeted therapies in routine care, with the final goal being to optimize pharmaceutical expenditure in oncology patients. Electronic Supplementary Materials Table S1 caption, which previously read: Biological drugs for cancer treatment available on the market, in the study period.

Should read:

Biological and non-biological targeted therapies for cancer treatment available on the market, in the study period.

## Electronic supplementary material

Below is the link to the electronic supplementary material.

Supplementary material 1 (PDF 223 kb)

