# Supplementary material for: Author Correction to: Prevalence of Use and Cost of Biological Drugs for Cancer Treatment: A 5-Year Picture from Southern Italy
Source: Clin Drug Investig. 2017 Dec 30;38(3):279–85. doi: 10.1007/s40261-017-0613-1 (PMC5834583; doi:10.1007/s40261-017-0613-1)
Supplement: Supplementary file 1 — Supplementary material 1 (PDF 223 kb) [file 40261_2017_613_MOESM1_ESM.pdf]

Clinical Drug Investigation

Prevalence of use and cost of biological and non-biological target therapies for cancer treatment: a 5 years' picture from Southern Italy

Simona Lucchesi* (1), Ilaria Marcianò* (2), Paolo Panagia (3), Rosanna Intelisano (3), Maria Pia Randazzo (4), Carmela Sgroi (5), Giuseppe Altavilla (6), Mariacarmela Santarpia (6), Vincenzo Adamo (6,7), Tindara Franchina (6,7), Francesco Ferraù (8), Gianluca Trifirò (2, 9, 10).

(1) Dpt. of Chemical Sciences, biological, pharmaceutical and environmental, University of Messina, Italy; (2) Clinical Pharmacology Unit, A.O.U. Policlinico "G. Martino", Messina, Italy; (3) A.O.U. Policlinico "G. Martino", Messina, Italy; (4) Papardo Hospital, Messina, Italy; (5) Pharmaceutical Department of Local Health Unit of Messina, Messina, Italy; (6) Medical Oncology Unit, Department of Adult and Childhood Human Pathology G. Barresi, University of Messina, Messina, Italy; (7) Medical Oncology Unit, Papardo Hospital; (8) Medical Oncology Unit, Hospital "San Vincenzo", Taormina, Messina, Italy; (9) Department of Biomedical and Dental Sciences and Morphofunctional Imaging, University of Messina, Italy; (10) Dpt. of Medical Informatics, Erasmus Medical Center, Rotterdam, Netherlands.

*First authorship shared

Corresponding author:
Gianluca Trifirò, MD, PhD
Department of Biomedical and Dental Sciences and Morphofunctional Imaging, University of Messina, Italy
Address: Via Consolare Valeria, Messina, Italy
Tel +39 0902213264
Fax +39 0902212711
Email: trifirog@unime.it

Table S1. Biological and non-biological target therapies for cancer treatment available on the market, in the study period.

ATC	Brand name	Active substance	Type of biologic	Indication for use	Biological target	AIFA postmarketing registry	Innovative drugs
L01XC02	Mabthera	Rituximab	MAB	-NHL<br>-CLL<br>-Rheumatoid arthritis<br>-Granulomatosis with polyangitis	CD20	Available for NHL, from 05/10/2009	
L01XC03	Herceptin	Trastuzumab	MAB	-Breast cancer (early and metastatic)<br>- Metastatic gastric cancer	HER2	For metastatic gastric cancer available from 14/01/2011	
L01XC04	Mabcampath	Alemtuzumab	MAB	-CLL	CD52	CLL	
L01XC05	Mylotarg	Gemtuzumab	MAB	-AML	CD33 positive		
L01XC06	Erbitux	Cetuximab	MAB	- mCRC<br>-Head and neck cancer (Advanced, recurrent or metastatic)	EGFR(HER1/ERBB1) (Kras wild type)	For mCRC Available from 02/10/2008;<br>For head and neck available from 23/12/2010	
L01XC07	Avastin	Bevacizumab	MAB	-mCRC<br>-Breast Cancer (metastatic)<br>-NSCLC (advanced, metastatic or recurrent)<br>-RCC (advanced or metastatic)<br>-Ovarian (advanced or recurrent) cancer<br>-Fallopian tube (advanced or recurrent) cancer	VEGFR	For mCRC available from 11/10/2005; -For breast Cancer, NSCLC and RCC available from 09/07/2008;<br>For ovarian, fallopian tube and peritoneal cancer available from 07/01/2014	

				-Cervical (persistent, recurrent or metastatic) cancer<br>-Peritoneal (advanced or recurrent) cancer			
L01XC08	Vectibix	Panitumumab	MAB	-mCRC	EGFR (Kras wild type)	Available from 17/01/2009	
L01XC09	Removab	Catumaxomab	MAB	-Malignant ascites	Epcam positive carcinomas	Available from 10/10/2011	
L01XC10	Arzerra	Ofatumumab	MAB	-CLL	CD20	Available from 14/06/2011	
L01XC11	Yervoy	Ipilimumab	MAB	-Melanoma (unresectable or metastatic)	CTLA4	Available from 09/03/2013	Important
L01XC12	Adcetris	Brentuximab vedotin	MAB	-HL (relapsed or refractory)<br>-ALCL (relapsed or refractory)	CD30	HL available from 08/07/2014	Potential
L01XC13	Perjeta	Pertuzumab	MAB	-Breast Cancer (metastatic or neoadjuvant)	HER2	Available from 08/07/2014	Important
L01XC14	Kadcyla	Trastuzumab emtansine	MAB	-Breast Cancer (unresectable, advanced or metastatic)	HER2	Available from 11/10/2014	Potential
L01XC15	Gazyvaro	Obinutuzumab	MAB	-CLL<br>-FL	CD20		
L01XE01	Glivec	Imatinib	TKI	-GIST (unresectable or metastatic)<br>-Dermatofibrosarcoma protuberans (recurrent or metastatic)<br>-Myelodysplastic / myeloproliferative disease	PDGF E SCF		

				- Advanced hypereosynotype syndrome (HES) and / or chronic eosinophilic leukemia (LEC)<br>- ALL (Ph+)<br>- CML (Ph+)			
L01XE02	Iressa	Gefitinib	TKI	-NSCLC (advanced or metastatic)	EGFR	Available from 11/06/2010	
L01XE03	Tarceva	Erlotinib	TKI	-NSCLC (advanced or metastatic)<br>-Pancreatic Cancer (metastatic)	EGFR	NSCLC from 28/07/2006	
L01XE04	Sutent	Sunitinib	TKI	-GIST(unresectable or metastatic)<br>-mRCC (advanced or metastatic)<br>-pNET (unresectable or metastatic)	PDGFR E VEGFR	mRCC from 04/10/2007	
L01XE05	Nexavar	Sorafenib	TKI	-Hepatocellular Carcinoma<br>-RCC<br>-Thyroid Carcinoma (advanced or metastatic)	VEGFR, PDGFR, KIT E RAF	For hepatocellular carcinoma available from 09/07/2008; -For RCC from 23/11/2006	
L01XE06	Sprycel	Dasatinib	TKI	-CML (Ph+)<br>-ALL (Ph+)	HER2(ERB2/neu), EGFR(HER1/ERBB1)	For all indications available from 26/05/2007	
L01XE07	Tyverb	Lapatinib	TKI	-Breast Cancer (advanced or metastatic)	EGFR(HER1/ERBB1), HER2 (ERB2/neu)	Available from 03/06/2009	
L01XE08	Tasigna	Nilotinib	TKI	-CML (Ph+)	ABL	Available from 08/08/2008	

L01XE09	Torisel	Temsirolimus	mTOR inhibitors	-RCC (advanced)<br>-Mantle cell lymphoma (relapsed or refractory)	mTor	For RCC available from 07/10/2008; For mantle cell lymphoma available from 25/08/2011	
L01XE10	Afinitor; Votubia	Everolimus	mTOR inhibitors	-Breast Cancer (advanced)<br>-Neuroendocrine tumors (of pancreatic, pulmonary or gastric origin)<br>-RCC (advanced)<br>- Subependymal Giant Cell Astrocytoma	mTor	For RCC available from 25/06/2010; for pancreatic tumors available from 21/12/2012; for breast cancer available from 21/07/2013	
L01XE11	Votrient	Pazopanib	TKI	-RCC (first line or advanced)<br>-Soft tissue sarcoma	VEGFR, PDGFR e KIT	Available for RCC, from 21/05/2011	
L01XE13	Giotrif	Afatinib	TKI	-NSCLC (Advanced or metastatic)<br>-NSCLC (Advanced or metastatic) squamous histology	EGFR (HER1/ERB1) e HER2(ERBB2/neu)	Available from 24/12/2014	
L01XE14	Bosulif	Bosutinib	TKI	-CML (Ph+)	ABL	Available from 01/10/2014	
L01XE16	Xalkori	Crizotinib	TKI	-NSCLC (advanced)	ALK, MET e ROS1 (ALK deletion or ROS1 gene alteration)	Available from 24/04/2013	
L01XE17	Inlyta	Axitinib	TKI	-RCC (advanced)	KIT, PDGFRβ, VEGFR1/2/3	Available from 05/01/2014	
L01XE18	Jakavi	Ruxolitinib	TKI	-Myelofibrosis<br>- Polycythaemia vera	JAK1/2	Available from 14/10/2014	
L01XE24	Iclusig	Ponatinib	TKI	-CML<br>-ALL (Ph+)	ABL, FGFR1-3, FLT3, VEGFR2 (T315I	Available for all indications from	

					mutation)	25/12/2014	
L01XE25	Mekinist	Trametinib	TKI	-Melanoma (unresectable or metastatic)	MEK (BRAF V600 mutation)		
L01XX32	Velcade	Bortezomib	Proteasome inhibitors	-MM<br>-Mantel Cell Lymphoma	PROTEOSOME	-For MM available from 23/07/2009	
L01XX44	Zaltrap	Aflibercept	VEGFR-Trap	-mCRC	PIGF, VEGFA/B	Available from 11/10/2014	
L03AC01	Proleukin	Aldesleuchin	Immunomodulatory agent	-MRCC			
M05BX04	Xgeva	Denosumab	MAB	-Bone metastases from solid tumor<br>-Giant cell tumor of the bone (unresectable)	RANKL	Bone metastases from solid tumor	
V10XX02	Zevalin	Ibritumomab tiuxetano	MAB	-Follicular lymphoma<br>-NHL (relapsed or refractory)	CD20	Available for follicular lymphoma, from 19/06/2005	

Legend: ATC= Anatomic Therapeutic Chemical classification system; TKI= Tyrosine kinase inhibitor; MAB= Monoclonal antibody; MTD= medullary thyroid carcinoma; NHL= Non Hodgkin's Lymphoma; HL= Hodgkin's Lymphoma; ALL= Acute Lymphoblastic leukemia; ALCL= Anaplastic Large Cell Lymphoma; CML = Chronic Mielogenous Leukemia; AML= Acute Myeloid Leukemia; CLL= Chronic lymphocytic leukemia; NSCLC= Non small Cell Lung Cancer; GIST = Gastrointestinal Stromal Tumor; RCC= Renal Cell Carcinoma; MRCC= Metastatic Renal Cell Cancer; MDT= Medullary Thyroid Cancer; MM=Myeloma multiple; mCRC= Metastatic colorectal; AIFA= Italian medicine agency

Table S2. Lower and upper bounds of 95% Confidence Intervals of prevalence of study drugs use, stratified by type of drug and calendar year.

	**95% Confidence Intervals bounds**									
	**mAb**		**TKi**		**Proteasome inhibitors**		**mTOR inhibitors**		**Overall**	
	**Lower**	**Upper**	**Lower**	**Upper**	**Lower**	**Upper**	**Lower**	**Upper**	**Lower**	**Upper**
**2010**	0,0561	0,0620	0,0366	0,0425	0,0143	0,0201	0,0079	0,0137	0,0700	0,0758
**2011**	0,0572	0,0631	0,0422	0,0481	0,0176	0,0235	0,0094	0,0153	0,0749	0,0808
**2012**	0,0688	0,0747	0,0484	0,0543	0,0204	0,0263	0,0075	0,0134	0,0874	0,0933
**2013**	0,0727	0,0786	0,0521	0,0581	0,0225	0,0284	0,0149	0,0208	0,0941	0,1000
**2014**	0,0783	0,0842	0,0508	0,0568	0,0242	0,0302	0,0230	0,0290	0,0995	0,1054

Legend: mAB= monoclonal antibodies, TKi= tyrosine-kinase inhibitors; mTOR= mammalian target of rapamycin.
